# The Relationship between Clinical and Psychophysical Assessments of Visual Perceptual Disturbances in Individuals at Clinical High Risk for Psychosis: A Preliminary Study

**DOI:** 10.3390/brainsci14080819

**Published:** 2024-08-16

**Authors:** Chloe Ifrah, Shaynna N. Herrera, Steven M. Silverstein, Cheryl M. Corcoran, James Gordon, Pamela D. Butler, Vance Zemon

**Affiliations:** 1 Ferkauf Graduate School of Psychology, Yeshiva University, Bronx, NY 10641, USA; vepman@aol.com; 2 Department of Psychiatry, Icahn School of Medicine at Mount Sinai, New York, NY 10029, USA; shaynna.herrera@mssm.edu (S.N.H.); cheryl.corcoran@mssm.edu (C.M.C.); 3 Department of Psychiatry, University of Rochester Medical Center, Rochester, NY 14642, USA; steven_silverstein@urmc.rochester.edu; 4 Department of Psychology, Hunter College, City University of New York, New York, NY 10065, USA; jgordon@hunter.cuny.edu; 5 Nathan S. Kline Institute for Psychiatric Research, Orangeburg, NY 10962, USA; pam.butler@nki.rfmh.org; 6 Department of Psychiatry, New York University School of Medicine, New York, NY 10016, USA

**Keywords:** schizophrenia, clinical high risk for psychosis, contrast sensitivity, perceptual disturbances, early-stage visual processing

## Abstract

This study investigated relations between a measure of early-stage visual function and self-reported visual anomalies in individuals at clinical high risk for psychosis (CHR-P). Eleven individuals at CHR identified via the Structured Interview for Psychosis-Risk Syndromes (SIPS) were recruited from a CHR-P research program in NYC. The sample was ~36% female, ranging from 16 to 33 years old (*M* = 23.90, *SD* = 6.14). Participants completed a contrast sensitivity task on an iPad with five spatial frequencies (0.41–13 cycles/degree) and completed the self-report Audio-Visual Abnormalities Questionnaire. Higher contrast sensitivity (better performance) to low spatial frequencies was associated with higher perceptual (*r* = 0.616, *p* = 0.044) and visual disturbances (*r* = 0.667, *p* = 0.025); lower contrast sensitivity to a middle spatial frequency was also associated with higher perceptual (*r* = −0.604, *p* = 0.049) and visual disturbances (*r* = −0.606, *p* = 0.048). This relation between the questionnaire and contrast sensitivity to low spatial frequency may be indicative of a reduction in lateral inhibition and “flooding” of environmental stimuli. The association with middle spatial frequencies, which play a critical role in face processing, may result in a range of perceptual abnormalities. These findings demonstrate that self-reported perceptual anomalies occur in these individuals and are linked to performance on a measure of early visual processing.

## 1. Introduction

There is significant heterogeneity in the clinical high risk for psychosis (CHR-P) state and course, and there has been a growing interest in improving the characterization of CHR-P individuals beyond clinical observations. CHR-P individuals are identified based on having the characteristic signs of the prodrome (i.e., the typically three- to four-year time period preceding the onset of threshold psychosis when individuals begin to develop subthreshold psychotic symptoms [1]) and are thus considered as being at elevated risk for developing a psychotic disorder. CHR-P syndromes include the experience of attenuated psychotic symptoms (APS), brief intermittent psychotic symptoms (BIPS), and genetic risk and deterioration syndrome (GRD). Recently, the European Psychiatric Association recommended the APS and BIPS criteria for use in psychotic disorder risk detection [2]. The genetic risk and functional decline (GRD) criterion of the CHR-P approach was not recommended due to a lack of evidence of risk prediction enhancement [2]. The DSM-5 Attenuated Psychosis Syndrome is a related condition listed as an “Other Specified Schizophrenia Spectrum and Other Psychotic Disorders” and “Conditions for Further Study” in the DSM-5. Individuals at CHR experience social, cognitive, and academic/vocational impairment, and are at risk for future development of threshold psychosis with approximately 20% of individuals at CHR developing psychosis within two years [3]. Perceptual processes are considered a key feature of schizophrenia and its risk states [4,5,6,7], and may serve as a biomarker for the CHR-P state and for risk of psychosis onset. 

Perceptual abnormalities are one of the most frequently endorsed APS in CHR-P individuals [8,9], and visual abnormalities/dysfunctions are thought to play a key role in the development of psychosis [5,6,10]. Many CHR-P individuals have reported anomalous perceptual experiences, including abnormal intensity of environmental stimuli, changes in the form, brightness, or depth of objects, feelings of being flooded and inundated, and the inability to focus attention on relevant details [11]. Studies have shown that these visual symptoms are especially pronounced in the initial stages of the illness before the evolution of the chronic state of schizophrenia [5]. A large body of literature also demonstrates that individuals with schizophrenia have deficits in laboratory measures of visual processing using psychophysical, electrophysiological, and neuroimaging approaches [12]. One measure of visual processing is contrast sensitivity (CS), which quantifies the ability of the visual system to distinguish objects from their background and is considered to be one of the most effective ways to isolate specific visual pathways [13]. CS deficits are consistently found in schizophrenia [13,14,15,16] and are associated with higher-level features of the illness such as impairments in cognition and functional outcome [17,18]. Surprisingly, individuals with a first episode of schizophrenia (unmedicated) showed elevated CS under select stimulus conditions [19,20]. Another study of first-episode cases that used different stimulus conditions and procedures, however, obtained contradictory results showing impaired CS [21]. 

To date, only one study has investigated both CS and perceptual anomalies in a CHR-P cohort. In 2007, Keri and Benedek [22] examined CS using a psychophysical task [23] with conditions designed to selectively tap transient (magnocellular) and sustained (parvocellular) parallel visual pathways [13,24,25] and a structured interview measure of perceptual anomalies (SIAPA) in 16 CHR-P individuals compared to 20 healthy controls. They found elevated CS in CHR-P compared to healthy controls in the condition that targets the magnocellular/transient pathway, which is maximally responsive to low luminance contrast and fast temporal changes, consistent with their findings in first-episode schizophrenia [19,20]. Kadivar et al. [21], again using different methods, found that CHR-P individuals had impaired CS. CHR-P individuals showed no differences in CS compared to healthy controls under the condition that targets the parvocellular/sustained pathway (maximally responsive to high luminance contrast and slow temporal changes). Visual SIAPA scores were positively correlated with CS in the “magnocellular/transient” condition, but not the “parvocellular/sustained” condition in CHR-P. The authors suggested that the high-risk state is associated with hyper-reactive transient pathways, which may be responsible for some anomalous visual perceptual experiences [22]. The goal of the current study was to extend this work by investigating CS, measured over a broad range of stimulus conditions, and compare it to a self-reported assessment of visual abnormalities in CHR-P. 

## 2. Materials and Methods

Participants were recruited from a pool of individuals who participated in other research studies at a CHR-P research program in New York City. The Structured Interview for Psychosis-Risk Syndromes (SIPS) [26,27] was administered to participants to characterize psychotic-like symptoms and determine the presence of a CHR-P syndrome. The SIPS has been widely used in CHR-P research for decades; it has strong psychometric properties, including good internal consistency, test–retest reliability, and validity [26,27]. Participants can meet criteria for one (or more) of the following psychosis-risk syndromes: (1) Attenuated Positive Symptom Syndrome (APSS), (2) Genetic Risk and Deterioration syndrome (GRD), and/or (3) Brief Intermittent Psychotic Symptom (BIPS). The SIPS also allows the assessor to make a determination for DSM-5 Attenuated Psychosis Syndrome. The SIPS consists of 19 items that use a 7-point severity scale to assess positive, negative, disorganized, and general symptoms (ranging from 0—absent to 6—severe). Attenuated psychotic symptoms are consistent with scores of 3–5 for individual positive symptom items. There are five positive (total score max = 30), six negative (total score max = 36), four disorganized (total score max = 24), and four general (total score max = 24) symptom items. 

Eleven participants who met criteria for a psychosis-risk syndrome on the SIPS completed the study. All eleven participants met criteria for Attenuated Positive Symptom Syndrome (APSS) and DSM-5 APS, as determined by the SIPS. CHR-P participants were English-speaking with normal (20/20) visual acuity tested with the S ETDRS 2000 Series Charts 1 and 2. Each eye was tested separately and together. The sample was ~36% female and the age range was 16–33 years (*M* = 23.90, *Mdn* = 23, *SD* = 6.14). This study was approved by the Icahn School of Medicine Mount Sinai Review Board (20-0018, approval 24 June 2020). All participants provided informed consent or assent if minors (with parental consent). 

Participants were administered the CS task on an iPad Pro [28]. Stimuli (horizontal sinusoidal grating patterns) were presented in an appearance/disappearance mode randomly to the left or right half of the screen within a uniform field of equal space-average luminance (100 cd/m^2^, frame rate = 60 frames/s). Spatial and temporal parameters of the stimuli were manipulated. For the purposes of the current work, five spatial patterns (gratings of the following spatial frequencies: 0.406, 1.626, 3.251, 6.502, and 13.005 cycles per degree [cyc/deg]) were presented for a duration of 500 ms during a forced-choice psychophysical task. The spatial frequency gratings were presented in an interleaved up–down-tracking procedure to obtain contrast thresholds (70.7% correct point on the psychometric function). In the two-alternative, forced-choice paradigm, participants indicated to the experimenter on which side of the screen the grating appeared by raising their right or left hand. The experimenter recorded the response by pressing buttons on the keypad. 

After the CS task, participants completed the Audio-Visual Abnormalities Questionnaire (AVAQ). The AVAQ is an 85-item self-report questionnaire that assesses abnormalities in auditory and visual processing [29]. The AVAQ (α = 0.99) consists of three subscales: visual, α = 0.98; auditory, α = 0.96; and audio-visual, α = 0.83). For the purposes of this study, only the total score and visual subscale were utilized. The highest total score a participant could achieve on the AVAQ was 255, with the visual subscale accounting for a possible 174 of the total (77%).

CS data were transformed (log_10_) for analyses [13,28,30]. Pearson correlations were computed between logCS values for each spatial frequency and the AVAQ scores (total and visual subscale score, AVAQVP). Bivariate scatterplots were created for all significant relationships to ensure that outliers were not driving the effects. Statistical tests were conducted to confirm that the data met the assumptions of linearity and normality of residuals.

## 3. Results

**Figure 1:**
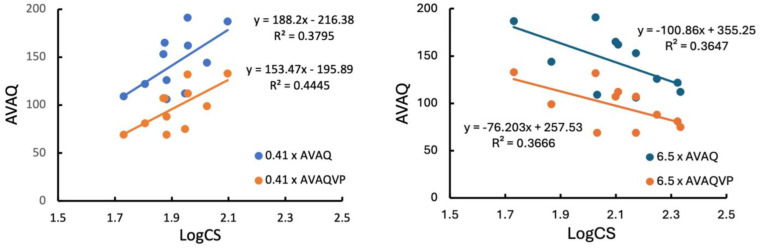
**Left Side**: LogCS/500 ms, 0.41 cyc/deg by AVAQ total score and AVAQ Visual Processing (VP) score. **Right Side**: LogCS/500 ms, 6.5 cyc/deg by AVAQ total score and AVAQ Visual Processing (VP) score. Blue lines are AVAQ total score and orange lines are AVAQ Visual Processing (VP) score.

LogCS to the lowest spatial frequency grating (0.41 cyc/deg) was positively and moderately correlated with the AVAQ total score (*r* = 0.616, *p* = 0.044, 95% bootstrapped CI [0.120, 0.877]) and AVAQVP score (*r* = 0.667, *p* = 0.025, 95% bootstrapped CI [0.164, 0.906]) (Figure 1, left side). In comparison, logCS to the 6.5 cyc/deg grating was negatively and moderately correlated with the AVAQ total score (*r* = −0.604, *p* = 0.049, 95% bootstrapped CI [−0.894, −0.125]) and AVAQVP (*r* = −0.606, *p* = 0.048, 95% bootstrapped CI [−0.912, −0.086]) (Figure 1, right side). These results indicate that a higher logCS to the low spatial frequency is associated with higher self-reported perceptual disturbances, especially visual disturbances, whereas a lower logCS to the middle spatial frequency is associated with higher self-reported perceptual disturbances, especially visual disturbances. No notable relations were observed between logCS and SIPS subscale scores. 

## 4. Discussion

CS has been studied in chronic schizophrenia with notable deficits reported in this population [13,14,15,16]. Given reports of the unexpected finding of enhanced CS in first-episode and CHR-P individuals [19,20,22], the current work assessed CS over a wide range of spatial conditions and investigated its relations with self-reported visual anomalies. Opposite effects were observed at a low and moderately high spatial frequency in that positive linear relations were obtained between AVAQ scores and logCS to a 0.41 cyc/deg grating, whereas negative linear relations were obtained between AVAQ scores and logCS to a 6.5 cyc/deg grating. 

The 0.41 cyc/deg and 6.5 cyc/deg conditions appear to bias responses toward the transient/magnocellular vs. sustained/parvocellular pathways, respectively [13,25,28,31]. If this is in fact the case, then our finding with the 0.41 cyc/deg condition is consistent with the results obtained using a magnocellular-biased condition with a CHR-P cohort [22]. In line with this finding, studies of first-episode individuals also demonstrated that increased CS to magnocellular-biased stimuli was related to increased visual perceptual anomalies [19,20]. Interestingly, Kelemen et al. [20] found that those individuals who later received antipsychotic medication no longer exhibited elevated CS. Perhaps the finding of elevated CS in the magnocellular-biased condition in these studies is indicative of a reduction in lateral inhibition, and lateral inhibition has been suggested to be the basis of the fall-off in contrast sensitivity at low spatial frequencies [32]. This alteration in CS might be a manifestation of a broader imbalance in excitation/inhibition. A prior modeling study has posited alternative mechanisms that could explain increased CS early in the course of schizophrenia, namely reduced retinal and lateral geniculate nucleus afferent activity, leading to overamplification at the cortical level [33]. This mechanism could account for the “flooding” of environmental stimuli that those with CHR-P often report. It should be noted that the above results differ from those of Kadivar et al. [21], who reported decreased CS under conditions that might reflect magnocellular function in CHR-P and first-episode cases. Kadivar et al. [21] used stimuli and techniques quite different from the previous work mentioned above and it is not clear what factors influenced this discrepancy in results. It has been hypothesized that there could be an illness-related process whereby the system shifts from hypersensitivity to hyposensitivity as the duration of illness increases following the onset of psychotic-like symptoms [21]. More research is needed to understand whether and when such a shift occurs and how it relates to self-reported visual anomalies.

This then leaves the question of why our CHR-P cohort showed a relationship between lower CS to medium and high spatial frequency information and increased visual anomalies. This may be due to the loss of critical spatial information required for functioning in the visual world. It has been shown that if a patient had decreased sensitivity at middle spatial frequencies, that patient was also likely to have a decreased ability to see faces, road signs, and commonplace objects [34]. Additionally, research on facial expression recognition has revealed that adults rely heavily on a range of middle spatial frequencies [35,36,37,38]. This provides one potential mechanism for findings of impaired facial affect recognition, facial perception, and reduced N170 amplitude (i.e., a component known for the structural encoding of a face) in those with CHR-P [39,40,41,42,43]. Additionally, studies in microelectronics workers indicate CS impairments that are specific to the middle spatial frequency range and neuropsychological deficits [44,45,46]. Taken together, one could understand how having a decreased logCS at middle spatial frequencies, which seems to be part of a general neurodegenerative process, could result in difficulty viewing the world and in a broad range of perceptual abnormalities.

Differences in the CS tasks between this study and the one of Keri and Benedek [22] may account for why we observed that higher CS at low spatial frequency but lower CS at medium to high spatial frequencies are both related to increased perceptual anomalies, whereas Keri and Benedek [22] only found a positive relationship under low spatial frequency conditions, but they did not test at spatial frequencies higher than 4 cyc/deg. Our finding of a relationship between lower CS and increased perceptual anomalies was at a higher 6.5 cyc/deg condition. Another difference between our study and that of Keri and Benedek [22] is that they utilized a structured interview to measure perceptual anomalies, whereas we utilized a self-report measure. 

There are several limitations in the current study including the small sample size, the lack of a comparison group, its cross-sectional design, and the risk of potential confounding factors that were not assessed, including substance use and comorbid diagnoses. This is a preliminary study that replicates a robust association between CS and visual anomalies in a new cohort, assessed using different measures.

## 5. Conclusions

Our findings extend previous work indicating that self-reported perceptual anomalies occur in help-seeking CHR-P individuals and that they are linked to performance on a psychophysical measure of early visual processing, contrast sensitivity. Low-level visual processes such as contrast sensitivity may yield a biomarker for psychosis as they appear to be highly related to self-reported visual symptoms among CHR-P individuals. Longitudinal studies are needed to characterize the trajectory of changes in contrast sensitivity throughout the course of the illness. Contrast sensitivity testing may lead to a greater understanding of the neural underpinnings of psychosis and advances in technology have increased the feasibility of assessing contrast sensitivity in the clinic [28].

## Data Availability

https://doi.org/10.6084/m9.figshare.26132938.v1, accessed on 14 August 2024.
